# Tele-expertise for diagnosis of skin lesions is cost-effective in a prison setting: A retrospective cohort study of 450 patients

**DOI:** 10.1371/journal.pone.0204545

**Published:** 2018-09-24

**Authors:** Kevin Zarca, Nathanael Charrier, Emmanuel Mahé, Fabien Guibal, Béatrice Carton, François Moreau, Isabelle Durand-Zaleski

**Affiliations:** 1 URC Eco Ile-de-France, AP-HP, Paris, France; 2 Department of Public Health, Henri Mondor-Albert Chenevier Hospitals, AP-HP, Créteil, France; 3 Department of Dermatology, Victor Dupouy Hospital, Argenteuil, France; 4 Department of Dermatology, Saint Louis Hospital and Université Paris Diderot, Sorbonne Paris Cité, AP-HP, Paris France; 5 André Mignot Hospital, Versailles, France; 6 Faculty of Medicine, University Paris-Est Créteil, Créteil, France; University of Naples, ITALY

## Abstract

**Context:**

The prevalence of skin diseases among prisoners is higher than in the general population. Diagnosing and treating these lesions require a dermatologic advice. A tele-expertise network in dermatology for prisoners including 8 health facilities in prison and 2 hospital dermatological departments was developed to improve access to dermatologists’ expertise in correctional facilities. Our objective was to evaluate the effectiveness and costs of tele-expertise in dermatology for prisoners.

**Methods:**

We carried out a retrospective cohort study on data collected by the information system of the tele-expertise network. We used the MAST (Model for ASsessment of Telemedicine) model to perform a multidimensional assessment including the proportion of patients with a completed treatment plan for the skin lesions, the proportion of technical problems, the quality of the pictures, the investment and operating costs and the satisfaction of the professionals.

**Results:**

Mean patient age was 34.2 years with 90% men. 511 requests for 450 patients were initiated. The delay from the connection to the tele-expertise software to the validation of the request was inferior to 7 min for 50% of the requests and inferior to 30 min for 85% of the requests. Overall, with tele-expertise, 82% of the patients had a completed treatment plan for the skin lesions, with 2.9% of all patients requiring a later face-to-face appointment or hospitalization, to be compared to a proportion of 35% of patients with a completed treatment plan when tele-expertise was not available. The most frequent lesions were acnea (22%) and atopic dermatitis (18%). The mean cost for one completed treatment plan was €184 by tele-expertise and €315 without tele-expertise. Tele-expertise was well accepted among physicians with all responders (n = 9) willing to continue using it.

**Conclusion:**

Tele-expertise is a dominant intervention in comparison to a face-to face consultation taking into account the cost of transportation and the proportion of canceled appointments and is acceptable for physicians.

**Trial registration:**

NCT02309905.

## Introduction

The prison population can be considered as a vulnerable group since disadvantaged social groups are overrepresented. Upon entrance, prisoners are in a weakened state of health due to poor access and utilization of health services and present a high prevalence of risky behavior [1]. In addition, there is a gradual aging of the prison population (the proportion of prisoners over age 60 increased from 1.0% in 1980 to 3.7% in 2014)[2], which can lead to a growing number of chronic conditions to be managed in prisons. Few articles have estimated the prevalence of skin diseases in the prison population [1], but previous studies in France confirmed a high need for dermatological consultations in prison [3,4].

Health care for prisoners in France is provided by medical units located inside the prisons. The physicians are usually general practitioners. Very few dermatologists provide medical consultations, mainly because medical resources are scarce in suburban areas in France where prisons are located and the hourly wage for working in prison is much lower than the fee for self-employed practitioners. When specialized dermatological advice is necessary, the patient is usually referred to a hospital for a medical appointment, accompanied by prison officers, inside armored cars [5]. If the patient is qualified as dangerous, an additional escort of army officers is required. To avoid any attempt of evasion, the prisoner is not informed of the date and time of the appointment. When it is time to leave the prison, more often than not, the prisoner cannot be found in their cell (e.g. out for a stroll, having other engagements such as meeting with their lawyer or relatives), and therefore the appointment–booked several weeks earlier—is canceled.

The median length of stay of prisoners in France is 4.5 months, and since the time to get an appointment with a dermatologist in hospitals varies between 2 and 3 months, most of the patients in need of secondary care are released without having seen the dermatologist. The situation therefore regarding the use of telemedicine in prisons is different from the general population where usual care is a face-to-face consultation whereas for prisoners the issue is the absence of care.

For the general population, teledermatology has been found successful in terms of diagnostic accuracy and reliability, access to care, clinical outcomes, and satisfaction [6,7]. Teledermatology could also increase the efficiency of the healthcare system by focusing the use of scarce dermatologists’ time on patients with complaints associated with greater risk of morbidity [8]. For underserved populations such as prisoners the potential benefits are even higher since the alternative is no access to specialist care [9].

A pilot study conducted in the medical unit of Fresnes prison [10] showed promising results and prompted a larger deployment of telemedicine for prisoners. Considering health in prison to be a strategic priority, the Paris Regional Health Agency *(Agence Régionale de Santé d’Ile-de-France)* funded a network including 8 primary care units in prisons (the local care units) and 2 dermatology units in hospital (the expert sites). The local care units were equipped with a telemedicine device allowing transmission of health information and images to the expert site *via* a secured server. Following the French typology of telemedicine [11], the purpose of the device was to provide tele-expertise for dermatological lesions, where a physician seeks the advice of one or more physician located at a remote site because of their particular training or skills, on the basis of medical information related to the care of a patient; rather than teleconsultation where the patient is present in front of the camera.

We evaluated the benefits of tele-expertise for diagnosis of skin lesions, specifically evaluating the clinical value of images in providing timely care and financial viability.

## Material and methods

### Population and data source

This study included all male and female patients over age 18 residing at 8 different prisons participating in the network of tele-expertise in dermatology, presenting to the medical units with a dermatologic condition for which a dermatologist advice is needed. Our database was a de-identified extraction from the information system of the telemedicine application, containing timestamps of each step of the workflow, description of the lesion and medical prescriptions. The following variables were recorded prospectively and extracted.

- Subject ID
- Sex
- Birth date
- Requesting site
- Expert site
- Timestamps of all the logs to the tele-expertise software of the requesting physician and the dermatologist
- Number of lesions
- Number of diagnosis
- Subsequent management needed
- Diagnosis (ICD-10)
- Number of photographs of the lesion
- Quality of the photographs

In order to have a control group, we collected data on requests for appointments to dermatologists consultation made in 2013 and 2014 in 1 prison of the network, for which tele-expertise was not implemented. These data were the date of the appointments and whether they were canceled or not.

10 physicians were involved in the study, 1 in each requesting center (n = 8) and 1 in each expert site (n = 2).

### Pre-intervention procedures

Prior to the intervention, the skin diseases were managed by the team members in the medical units. For cases where a dermatologist advice was necessary, the patients needed to be transferred for hospital consultation or hospitalization.

### Intervention

The teledermatology platform was implemented by the SESAN group (http://www.sesan.fr/) which provides a solution for the deployment of telemedicine in the Paris Region. Physicians, both in requesting and expert sites were trained by the project management and IT teams on how to use the tele-expertise platform. Physicians of the local care units were trained by dermatologists of the expert sites on how to properly photograph skin lesions (full body and close-up detail for the lesions). 2 dermatologists were in charge of the tele-expertise in the 2 expert sites, one dermatologist for each site. An agreement was signed beforehand between hospitals and prisons to define the correspondent dermatologists of a given medical unit in prison.

The physicians of the local care unit took one or several pictures of the skin lesions, and copied these to their computer from which they then connected to the tele-expertise platform, and created a record with clinical details and selected pictures. They then validated the record, and the record was immediately uploaded to the server.

**Fig 1:**
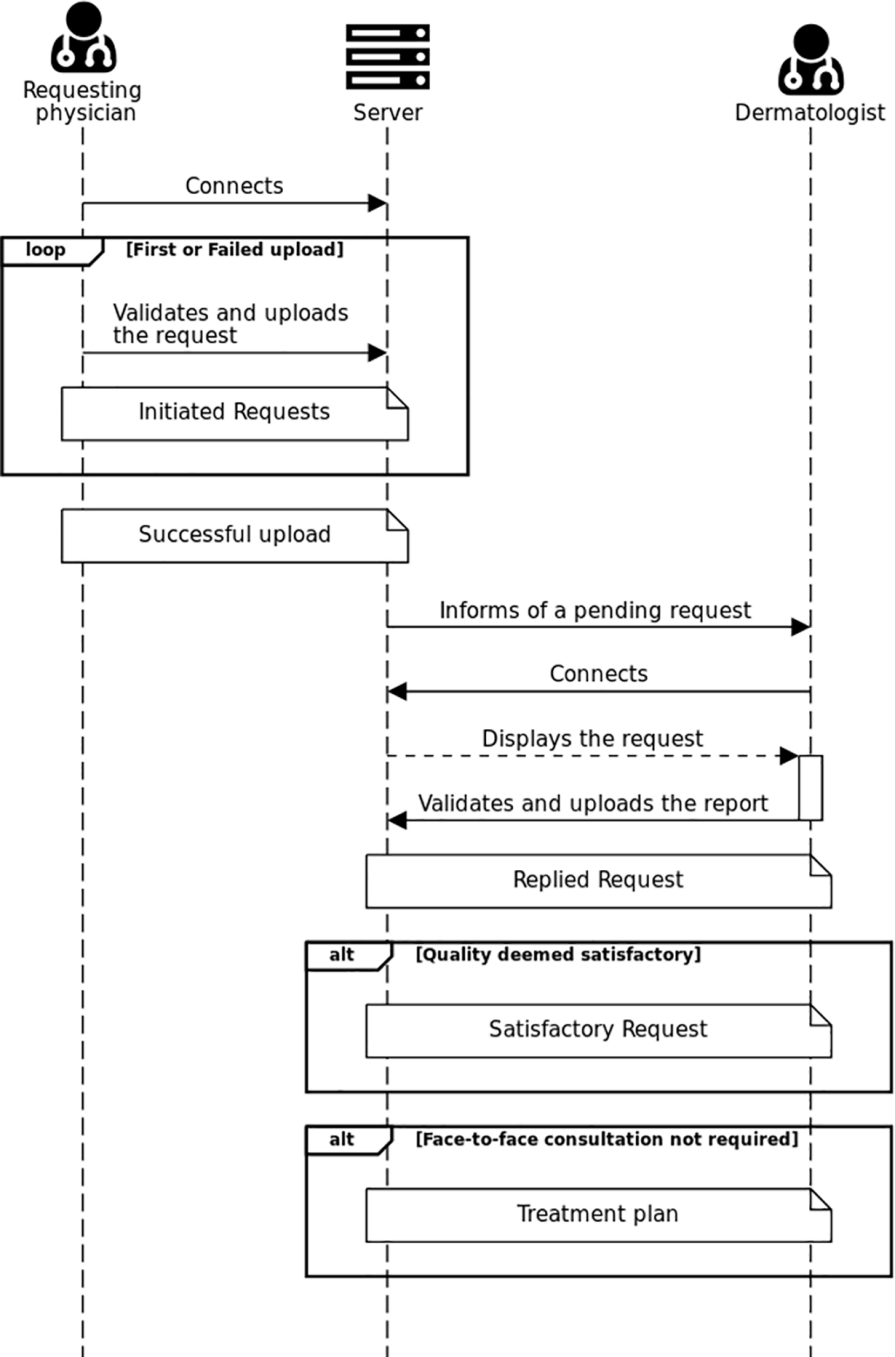
Simplified sequence diagram of the tele-expertise procedure. loop: The bordered part of the diagram restarts as long as the condition is true. alt: The bordered part of the diagram occurs only if the condition is true.

Once the record was uploaded, the dermatologist was informed by email that a patient was waiting for his expertise. The dermatologist then connected to the platform. After analysis, the dermatologist wrote a report with the treatment plan (Fig 1).

#### Definitions

**Initiated requests:** All requests initiated by the requesting physician. For the group without tele-expertise, it corresponds to the appointment to the dermatologist. For the group with tele-expertise, it corresponds to all requests made by tele-expertise, whether a technical problem occurred or not.

**Successful upload:** all initiated requests successfully uploaded to the tele-expertise server.

**Replied request:** all successfully uploaded requests with a reply of the dermatologist.

**Satisfactory request:** all replied requests for which the dermatologist considered the quality of the images satisfactory.

**Completed treatment plan:** for the group without tele-expertise, it corresponded to a face-to-face consultation occurring with a dermatologist or a hospitalization in a dermatology unit. For the group with tele-expertise, it corresponded to a satisfactory request for which a face-to-face consultation was not deemed mandatory by the dermatologist or the patient was transported for a face-to-face consultation or for an hospitalization in a dermatology unit.

Successful upload, replied requests and satisfactory request are only applicable for the intervention group.

### Study design and endpoints

We carried out a 1-year cohort study, from June 2014 to June 2015 and used the MAST model [12] to perform a multidimensional assessment. MAST is a framework for assessing the value of telemedicine that is based on the core health technology assessment model [13]. We collected results on safety, clinical effectiveness, economic aspects and organizational aspects.

The primary endpoint was the proportion of patients with a completed treatment plan for the skin lesion.

Other endpoints were the proportion of non-response to a request among all initiated requests, the delay from the successful uploaded request to the response among all replied requests, the duration of the case analysis and the medical report edition among all replied requests and the number of face to face appointments.

We studied the quality of the lesion pictures assessed by the dermatologist as being very satisfactory, satisfactory or unsatisfactory, but did not measure diagnostic accuracy.

We adopted a collective perspective, including the cost for the prison (transport), the cost for health facilities inside prison and the hospital costs. We estimated the cost of one request leading to a completed treatment plan.

We measured the investment costs and the operating cost. The investment cost was the amount of money paid by the health facilities to the project management and IT Teams and it included the price of a camera and a dermatoscope in every local care unit.

The operating costs covered annual cost of maintenance of the tele-expertise network and the human resource needed to produce the tele-expertise consultation. Human resource was valued by a bottom-up approach to measure durations for health providers, using the hospital salary cost and by a publicly available report on transportation costs in prisons [14].

We used an amortization on a 5-year duration and a 4% discounting rate, as specified in French Health Authorities guidelines [15], and realized sensitivity analyses including: amortization (2–10 years), discounting rate (0%-6%), duration of the request creation (10–50 min) and the number of local care units (2–10) with a baseline of 50 annual tele-expertise per site.

A satisfaction survey was submitted by email to all the physicians in the network (n = 10) in December 2015, 6 months after the end of the study period. They were still using tele-expertise at that time. The survey was adapted from the MAST questionnaire [16], scored using a six-item Lickert scale.

### Assumptions for cost analysis

The cost analysis was based on conservative hypotheses as follows:

- Without tele-expertise, when the appointment was not followed by a consultation, the cost for the prison was assumed to be zero.
- When the picture quality was not satisfactory, another tele-expertise was carried out.
- Only prison health facilities paid the telemedicine investment and operating costs. Not the expert site.
- The proportion of patients transported when required is the same in both groups.

### Ethics

This study was reviewed by an institutional review board (CCTIRS: Comité consultatif sur le traitement de l'information en matière de recherche; approval 15.928) and obtained the authorization CNIL 1881323. Data being unidentified, this study did not present potential risks to individuals or individual privacy.

## Results

### Efficacy and safety

**Fig 2:**
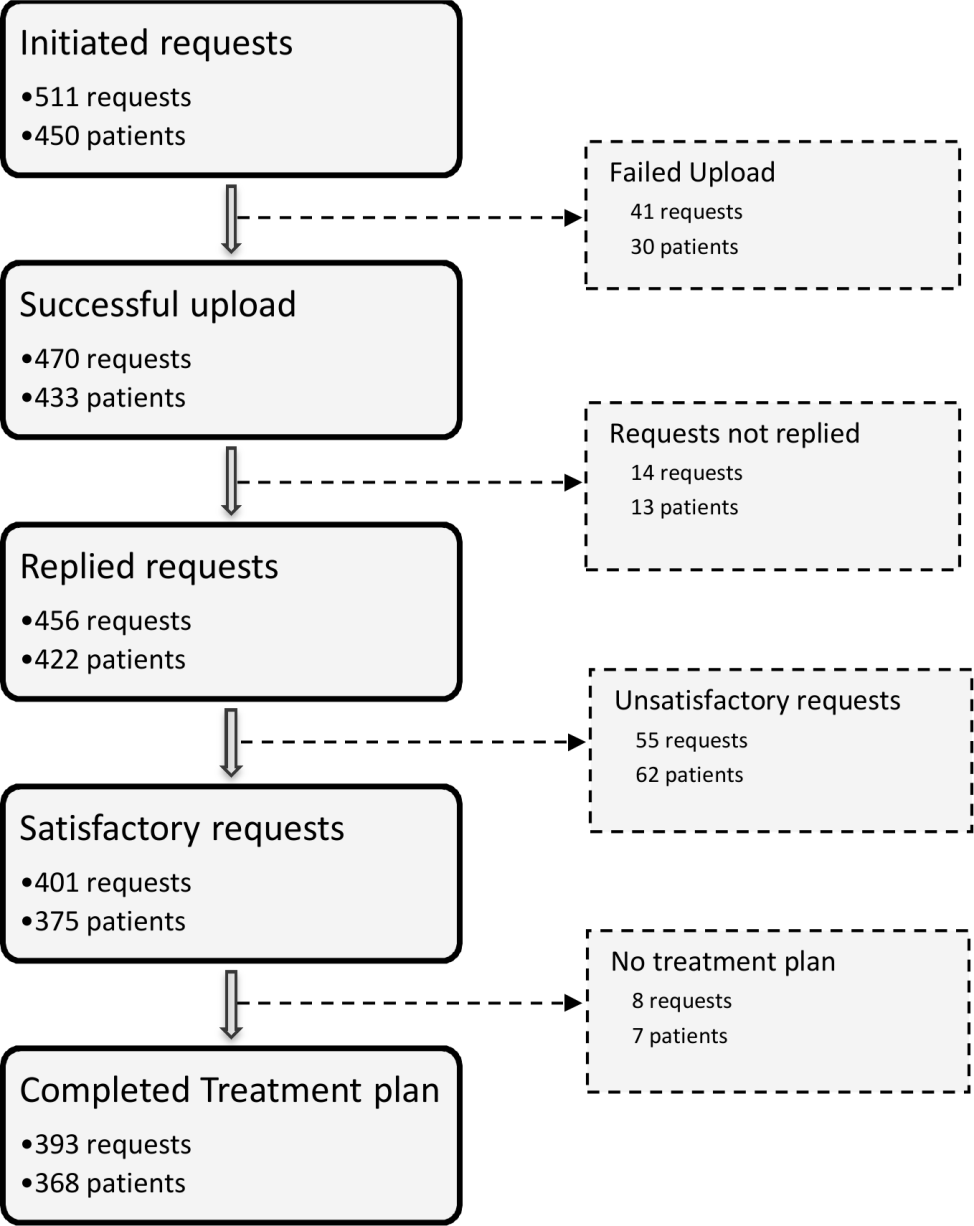
Flowchart of the requests. Multiple requests could be performed for one patient.

511 requests were initiated for 450 patients (Fig 2).

Mean patient age was 34.2 years, with a proportion of 90% of men.

The median delay for obtaining a response was 5.0 days (Table 1).

**Table 1:** Description of the distribution of the duration of major steps of the tele-expertise.

Durations (days)	Mean (sd)	Range	Median[IQR]
Overall duration, from the Request initiation to the Response	8.8 (13.4)	0.0–70.9	5.0 [0.2–9.1]
Delay between medical case upload to agreement to review the case by a dermatologist	7.9 (12.0)	0.0–64.0	4.0 [0.2–8.5]

For the requesting physician, the duration from the connection to the tele-expertise software to the validation of the request was inferior to 7 min for 50% of all the initiated requests and inferior to 30 min for 85% of them.

For the dermatologist, the duration of the case analysis from the connection to the medical report edition was inferior to 6 min for 50% of the requests and inferior to 30 min for 90% of the requests.

82% (375/456) of the replied requests contained only one lesion to analyze, 13% two lesions, and 4% over two lesions. 75% of lesions were classified following the ICD-10 as “Diseases of the skin and subcutaneous tissue”, 14% were classified in “Certain infectious and parasitic diseases”. The most frequent lesions were acne (22%) and Atopic dermatitis (18%). One melanoma was diagnosed during the study.

With regards to the picture quality, 40% of the replied requests were satisfactory and 48% were very satisfactory.

For 2.9% (11/375) of patients for which the request was satisfactory, the recommended management of the patient was a face-to-face consultation or a hospitalization for further investigation. Among them, 7 patients could not be transported, cases for which the treatment plan for the skin lesion could not be completed with tele-expertise.

Overall, 82% (368/450) of the patients for which the request was initiated by tele-expertise had a complete treatment plan.

In the control group, 54 initiated requests (appointments) were programmed, among which 35% led to a completed treatment plan (i.e. the face-to-face consultation or hospitalization finally took place).

### Costs

For 8 equipped sites, the total investment cost was €102,286. The subscription cost for the software and the data storage into secured servers was €25,920 per year for all 8 sites.

The median duration of a tele-expertise request was 23 min from the entrance in the examination room to the successful upload of the record. That corresponds to a human resource cost of €36 for the local care unit, taking account the unsuccessful uploads, unanswered requests and unsatisfactory requests. For the expert site, the median duration of the analysis of the record was 6 min, corresponding to €7. The mean transport cost was €8 (the product of the cost of transportation of prisoners (€900) and the proportion of prisoners transported (4/450)).

The average cost of human resources in the tele-expertise arm was estimated to be €51 for one completed treatment plan. Without tele-expertise, the cost of one completed treatement plan was €315 (the product of the cost of transportation of prisoners and the proportion of prisoners transported (0.35)).

**Fig 3:**
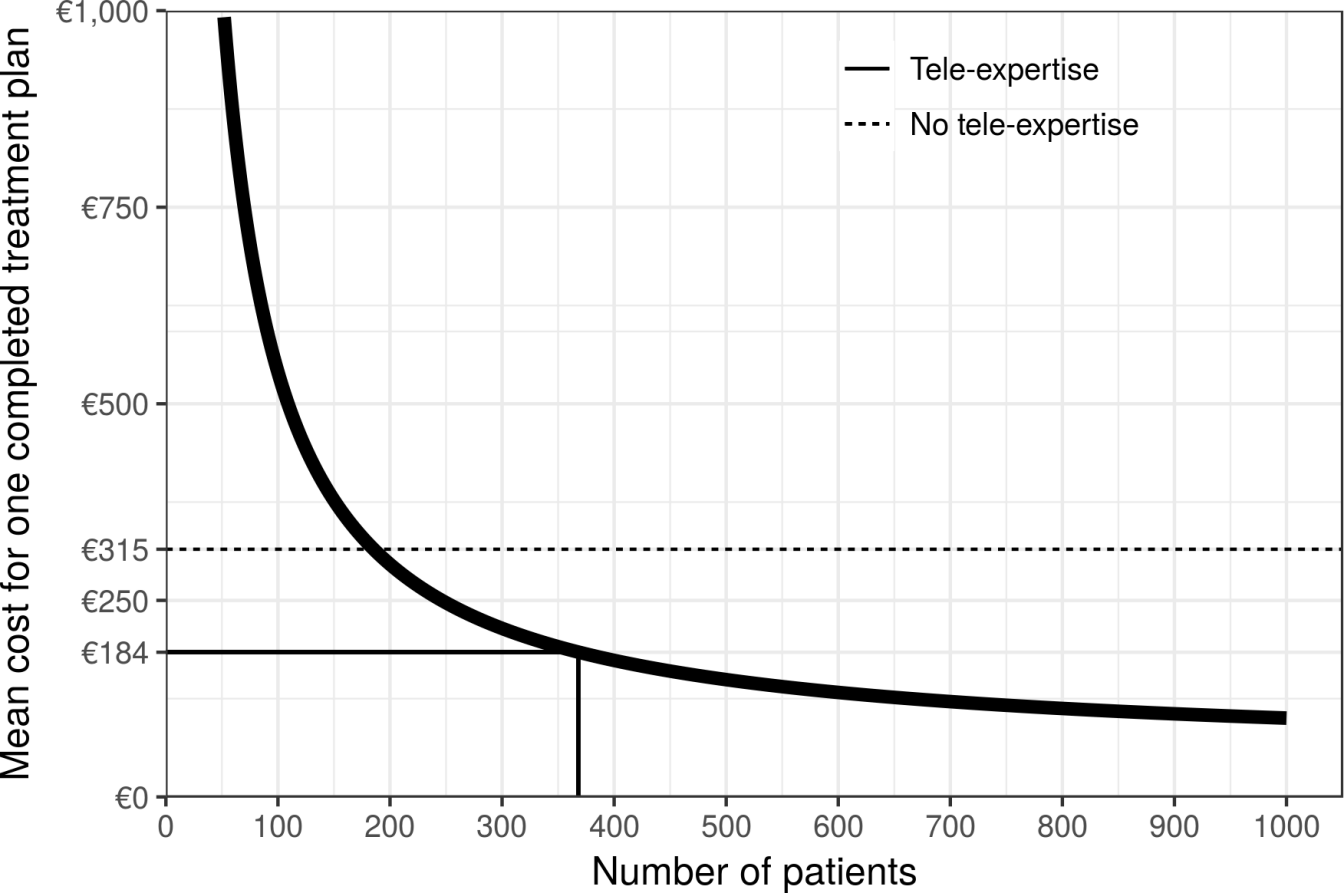
Mean cost for one completed treatment plan depending on the number of patients. As investment costs are substantial and fixed, economies of scales are possible in the tele-expertise group. As soon as the number of annual patients reaches 186, the mean cost of one completed treatment plan equals the cost without tele-expertise.

The total mean cost for one completed treatement plan depends on the number of patients (Fig 3). For 368 patients every year and 8 sites, the mean cost is €184 / completed treatment plan distributed as follows: 34% investment, 66% operating cost (30% human resource, 36% software).

**Fig 4:**
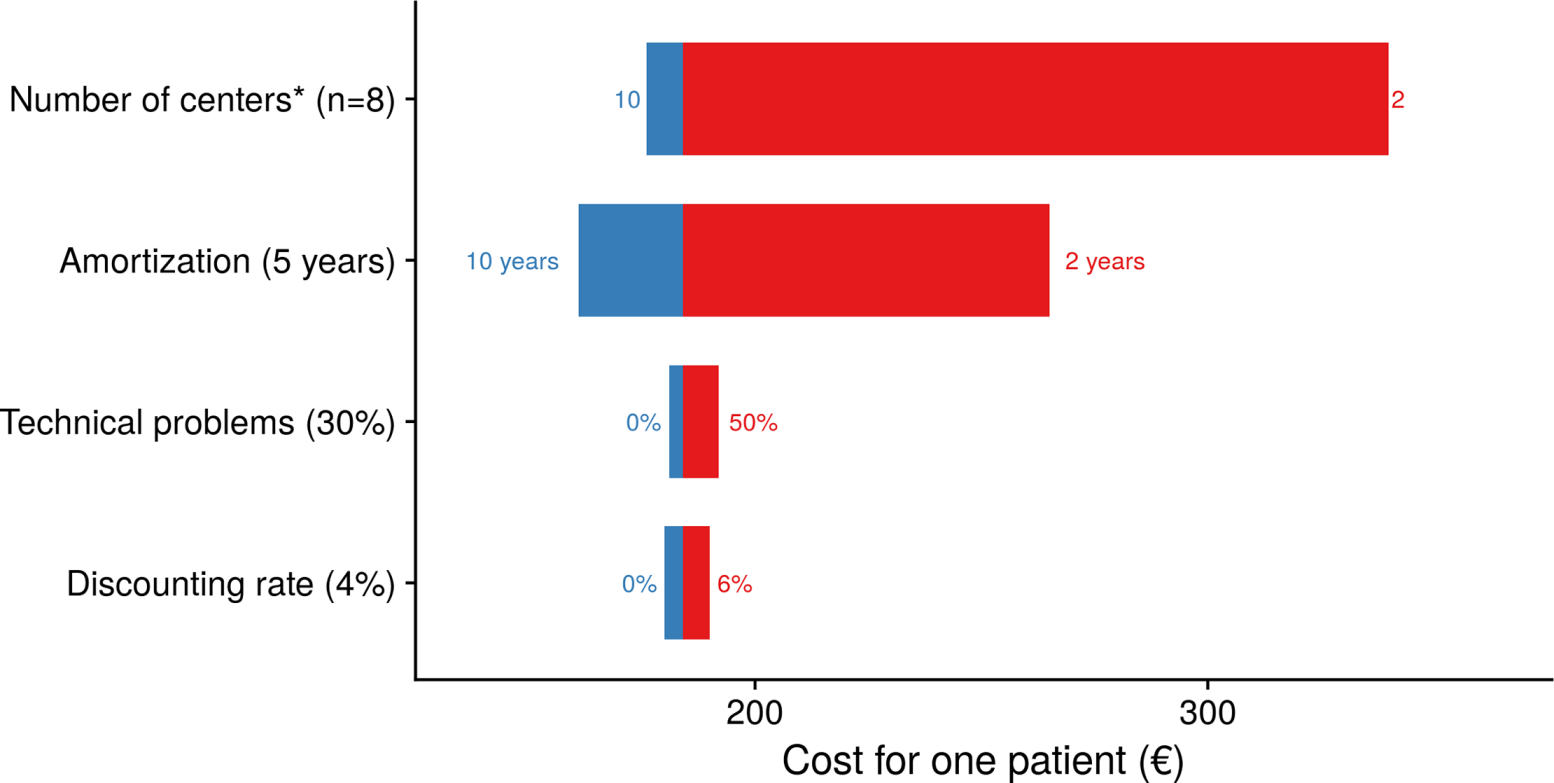
Tornado diagram of the sensitivity analysis of the mean cost for one completed treatment plan. Costs determinants are listed vertically, ordered so that the largest bar (the most sensitive data) appears at the top of the chart, and the smallest bar at the bottom. The baseline is €184. *With 50 satisfactory requests at each site.

In our sensitivity analysis, the main parameter was the number of local care units, the total cost varying between €168 and €335: (Fig 4).

### Satisfaction of professionals

9 physicians completed the form, 7 being physicians for the local care unit, and 2 for the expert sites. Of these 9 physicians, 7 fully agreed with the affirmation: “You would like to continue to use the telemedicine service” and the remaining being rather in agreement.

## Discussion

Tele-expertise in dermatology for prisoners allowed a higher proportion of patients with a completed treatment plan for the skin lesions (82% vs. 35%). For a total of 511 annual requests for 450 patients from 8 prison health centers and with 2 dermatological centers carrying out the analysis, the cost of one completed treatment plan was €184. We found that tele-expertise was therefore a dominant intervention in comparison to the cost of an appointment taking into account the cost of transportation and the proportion of canceled appointment (€315). Furthermore the physicians using tele-expertise seemed satisfied with the new workflow.

Dermatology has been actively studied in telemedicine recently [7,17], but few authors conducted a cost analysis evaluation [18–20]. Our study is the first to assess the effectiveness of tele-expertise in dermatology for the prisoners.

The high proportion of patient with a completed treatment plan using tele-expertise compared to the face-to-face consultation can be explained with the grid provided by Landow et al [21]: although there was no preselection of the patients, a) the photographic images were high quality, b) a dermatoscope was used and c) in prison, as the patients are captive, implementing teleconsultation recommendations is easier than in the general setting, without necessity to require to a follow-up face-to-face visit to the dermatology clinic.

For an annual number of patients of 186 or more we showed that the cost of tele-expertise was less than without tele-expertise. Below that number, the incremental cost-effectiveness ratio may remain acceptable, depending on the decision-maker threshold.

Results of the analysis can be considered robust, since the intervals of the performed sensitivity analyses were narrow.

This work has limitations: Our control group was made of only one prison and may not be representative of the situation in all prisons. However, this prison had a similar capacity as the prisons in the control group, similar demographics, length of stay and reasons for detention [22]. We could not evaluate the diagnostic accuracy of telemedicine, nor the inter-observer concordance but assumed from other publications on teledermatology that it would be satisfactory [7]. We could not rigorously analyze the causes of "failed upload, unanswered requests and unsatisfactory requests" because this information was not recorded in the database. However discussions with dermatologists indicated that failed uploads were mostly due to a network or server issue, unanswered requests mostly due to graphical user interfaces problems (record not displayed), and unsatisfactory requests to bad photograph quality.

We were also unable to measure patient satisfaction because the length of stay for most prisoners is shorter than the length of study.

The delay to get a response by tele-expertise may seem high (median: 5.0 days), but, since dermatology is a specialty that usually does not require emergency appointment, the physicians did not consider it as an issue.

One stated objective of tele-expertise was to reduce the need for the usual procedure of prisoner transportation to hospital, i.e. better targeting of patients needing to be transferred from prisons to hospitals. Other, more efficient organizations might be worth comparing. The weekly or monthly visit of a dermatologist to the prison could possibly be sufficient to improve access to the dermatology expertise of the prison population at an acceptable cost, however the scarcity of dermatologists who volunteered to work in prisons’ hospitals rendered this option unattractive.

We were unable to assess whether tele-expertise led to an increase of requests which could have been diagnosed and treated without the need for a dermatologist advice. We can hypothesize that there is a learning curve in the use of tele-expertise. The general practitioners working in hospital prisons were given the possibility of near-instant dermatologist’s backup for their diagnoses of skin lesions: while at first that possibility would increase requests, the confirmatory answers provided by dermatologists would over time increase the confidence of GPs in their own diagnoses. Other patients who present complex dermatological cases outside prisons could also benefit from tele-expertise, however this would require decentralized platforms in doctors’ offices which is currently too high an investment to consider. This would be different however for medical homes (multidisciplinary clinics) that treat a large patient volume.

Our study focused on skin diseases but any expertise with a high activity in prison which traditionally requires a face-to-face consultation and lacking specialists willing to work in prisons might give similar results. We also expect increased efficiency of the tele-expertise, through the learning curve of computer skills of the physicians and technical improvements in the platform.

## Conclusion

Tele-expertise for diagnosis of dermatologic lesions among prisoners was found to be effective, increasing the proportion of patients with a completed treatment plan and the overall satisfaction of the requesting physicians at a cost far lower than a dermatologists’ consultation in a hospital.
